# Genius and Insanity

**Published:** 1895-06-08

**Authors:** 


### Genius and Insanity.

Cesare Lombroso, a distinguished professor at Turin, came to the conclusion, some years ago, that there were two classes of criminals. The one set were not different to other men. Confronted by some sudden temptation, overborne by a gust of passion, they had committed their fault, and, after due expiation, unless corrupted by prison surroundings or driven into new crime by uncharitable persecution, they returned to the honourable pursuit of ordinary avocations. But in the other set, the tendency to crime is inherent; born with them at their birth, and not to be effaced by education or by punishment. Such persons show certain remarkable physical and mental characters. In the form and shape of the head, in the features, and even in the bones and nerves they show physical resemblance to the lower races. Their eyesight, their hearing, and their sense of touch is less acute; their mental faculties are duller; their sensibility to pain and pleasure is benumbed. Lombroso explains the existence of these by supposing that they are degenerate; that they have "thrown back" to a more primitive condition of the human race, as when in a box of garden seedlings a few are found to have lost the colour and shape produced by the careful selection of the florist, and to have reverted to the wild form as it was before the florist began to improve the race by selection. Carrying out this idea of the occasional occurrence of degenerate types among men, Lombroso was led to the conclusion that men of genius generally show degenerate characters akin to those found among the insane.

Max Nordau, following in the footsteps of Lombroso, has written a most interesting book, in which he tries to show that a wave of degeneracy has been spreading over Europe, and that most of the famous men that we have admired in the last fifty years are more or less degenerate and insane. He thinks that the restless conditions of modern life, the high pressure at which we live, the jars to the system given by railway travelling, all the unnatural conditions of our modern civilisation, are ruining the nervous system of the race. But the signs of degeneration are most conspicuous among those whom we have regarded as heaven-born geniuses. Everyone knows of the madness of Poe and Baudelaire, his French translator and imitator, and of Swift, and Rousseau, and Gogol. But it will surprise most of us to read that men like Ruskin and Ibsen, Wagner and Tolstoi, exhibit many characters in common with the higher types of the degenerate.

Thousands of years ago, in the dawn of civilisation, when hieroglyphics were invented, they were employed as rude symbols to convey ideas. Ruskin has reverted to this primite conception, and has taught that paintings are meant first of all to convey ideas, especially religious ideas. And so he persuaded the English people to see beauty in the rude art of the early Italians, simply because there was the presentation of religious conceptions in the distorted drawing and in the crude and faded colours of their rotting canvases. Ruskin's possession of lofty religious thought must not blind us to the craziness of his ideas, for religious emotion not seldom accompanies even acute mania.

By a careful analysis of his works, Nordau attempts to show that Ibsen is a mystic degenerate, his mind overborne by "theological obsessions of original sin, of confession and redemption," with the result that such ideas continually recur in a crazy and distorted fashion. But he exhibits in his attacks on society precisely the frame of mind that Lombroso found in the degenerate criminal, "in consequence of his neurotic and impulsive nature, and his hatred of the institutions which have punished or imprisoned him, he is a perpetual latent political rebel." Ibsen, however, is only a theoretical criminal, his motor centres not being powerful enough to transmute his anarchically criminal ideas into deeds. But the most curious feature in which the writings of Ibsen display his degenerate nature is his treatment of women. Nordau analyses what many people have regarded as Ibsen's chief triumph, his notion that women should abandon, home and children and over-ride all their natural instincts to "live their own lives." He shows that there is a close parallelism between Ibsen's views and the sexual perversion of a class of degenerates that has been studied and described in Continental asylums.

Richard Wagner, the founder of a new school of music, described by enthusiasts as the "music of the future," combined in his character a large number of the signs of degeneration. He was afflicted with the persecution mania, megalomania, and mysticism. His writings display signs of graphomania, incoherence, fugitive ideation, and a tendency to idiotic punning. "As with all morbid erotics, woman presents herself to him as a terrible force of nature, of which man is the trembling, helpless victim." The great theory running through his musical composition, the presence of an unending melody, is merely an atavistic or degenerate return to primitive barbaric music.

It has been possible only to indicate in a very brief fashion some of the chief points in Max Nordau's remarkable book. All who read it will find it to be a profound attack upon the grotesque and bizarre degenerate fancies which many have been accustomed to regard as the works of genius in the nineteenth century.

"Degeneration." By Max Nordau. Translated from the second German edition. (London: Heinemann. 1895.)

